# A five‐day‐old child with lipid hemihypertrophy: A case report

**DOI:** 10.1002/ccr3.8476

**Published:** 2024-02-07

**Authors:** Kayvan Mirnia, Maryam Saeedi, Razieh Sangsari, Kimia Kazemzadeh

**Affiliations:** ^1^ Division of Neonatology, Department of Pediatrics, Children's Medical Center, Pediatric Center of Excellence, Faculty of Medicine Tehran University of Medical Sciences Tehran Iran; ^2^ Students' Scientific Research Center Tehran University of Medical Sciences Tehran Iran; ^3^ Network of Neurosurgery and Artificial Intelligence (NONAI) Universal Scientific Education and Research Network (USERN) Tehran Iran

**Keywords:** asymmetry, hemihyperplasia, hemihypertrophy, lipid hemihypertrophy, lipomatosis

## Abstract

**Key Clinical Message:**

Lipid hemihypertrophy should be considered in the differential diagnosis of neonatal asymmetry. Early recognition and further evaluation for associated disorders are important for appropriate management and surveillance of potential complications.

**Abstract:**

We present the case of a 5‐day‐old female neonate who presented with a visibly enlarged right thigh, right labia majora, and below the right mandible. This case report highlights the importance of early identification, comprehensive evaluation, and multidisciplinary management in neonates with lipid hemihypertrophy to optimize their long‐term outcomes and quality of life.

## 1 INTRODUCTION

Asymmetry of the body has captivated the attention of artists and sculptors from the classical periods of Greece and Rome. Meckel initially referenced congenital hemihypertrophy in 1822, while it was Wagner who provided the first detailed characterization of this affliction in 1839.^1^ Additionally to the apparent aesthetic and orthopedic difficulties that emerge with hemihypertrophy, it is recognized to be associated with a variety of intra‐abdominal neoplastic and non‐neoplastic conditions.^2^

The prevalence of mild asymmetry between the two sides of the body is widespread, not always conspicuous, and ordinarily necessitates precise quantification. Hemihypertrophy is optimally characterized as the asymmetry between the two sides of the form to a greater extent than can be attributed to typical fluctuation. Arbitrarily, the growth discrepancy must be greater than 5%.^3^ This could encompass distinctions in the length or circumference. The hemihypertrophy may encompass the whole embryonic tissues (total) or solely one tissue (limited). It may impede one moiety of the body (complex), one appendage (segmental) or a digit, a mammary gland, or one side of a phallus (simple). It may reveal itself in a crossed or unilateral manner.^4^, ^5^

Herein, we aim to present a case of a 5‐day‐old neonate with lipid hemihypertrophy, also known as lipomatosis. Lipomatosis is a pathological condition characterized by an inadequately defined overgrowth of mature adipose tissue that proliferates in an invasive manner and encompasses substantial regions of an extremity, face, or trunk.^6^

## 2 CASE PRESENTATION

A 5‐day‐old female was referred to the Children's Medical Center, due to the chief complaint of presenting with asymmetry, which had been present since birth. This patient had been formerly admitted to a different hospital due to the presence of genital ambiguity and respiratory distress. Additionally, the neonate demonstrated jaundice from the third day post‐partum. Neither poor feeding nor fever were reported. The physical examination revealed the presence of hypertrophy in the right thigh, right labia majora, and below the right mandible, with no tenderness elicited upon palpation. It was noted that the gestational age of the infant was 38 weeks and 4 days and that the vaginal delivery was prolonged. This neonate was the first child of the family and the mother had no history of significant medical conditions, gestational diabetes, or pre‐eclampsia.

A paraclinical examination was requested for the patient. The ultrasonography indicated the presence of edema and heightened intracutaneous adipose tissue in the region of edema of the right labia major, medial surface of the right thigh, and right portion of the neck. No vascular or pathological lesions were detected. Moreover, the uterus was noted to be in a normal echo and size, situated in its appropriate anatomical location. The radiography demonstrated normal osseous density and standard length of the vertebral column and sacrum. See the results of other paraclinical tests on day 5 after birth in Table 1.

**TABLE 1:** Paraclinical tests on day 5 after birth.

Blood glucose	BUN	Creatinine	Calcium	Phosphorus	Total protein	Albumin	G6PD	Bilirubin total	Bilirubin Direct	Magnesium	CRP	WBC	RBC	Hb	PLT	MCV	MCH	MCHC
82 mg/dL	6 mg/dL	0.2 mg/dL	8.2 mg/dL	3 mg/dL	4.4 g/dL	3.5 g/dL	20 U/gHb	20 mg/dL	0.6 mg/dL	2.4 mEq/L	1 mg/dL	10.57 10^3^/μl	4.92 10^6^/μl	16.8 g/dL	232 10^3^/μl	100.2 fL	34.1 pg	34.1 g/dL

**FIGURE 1:**
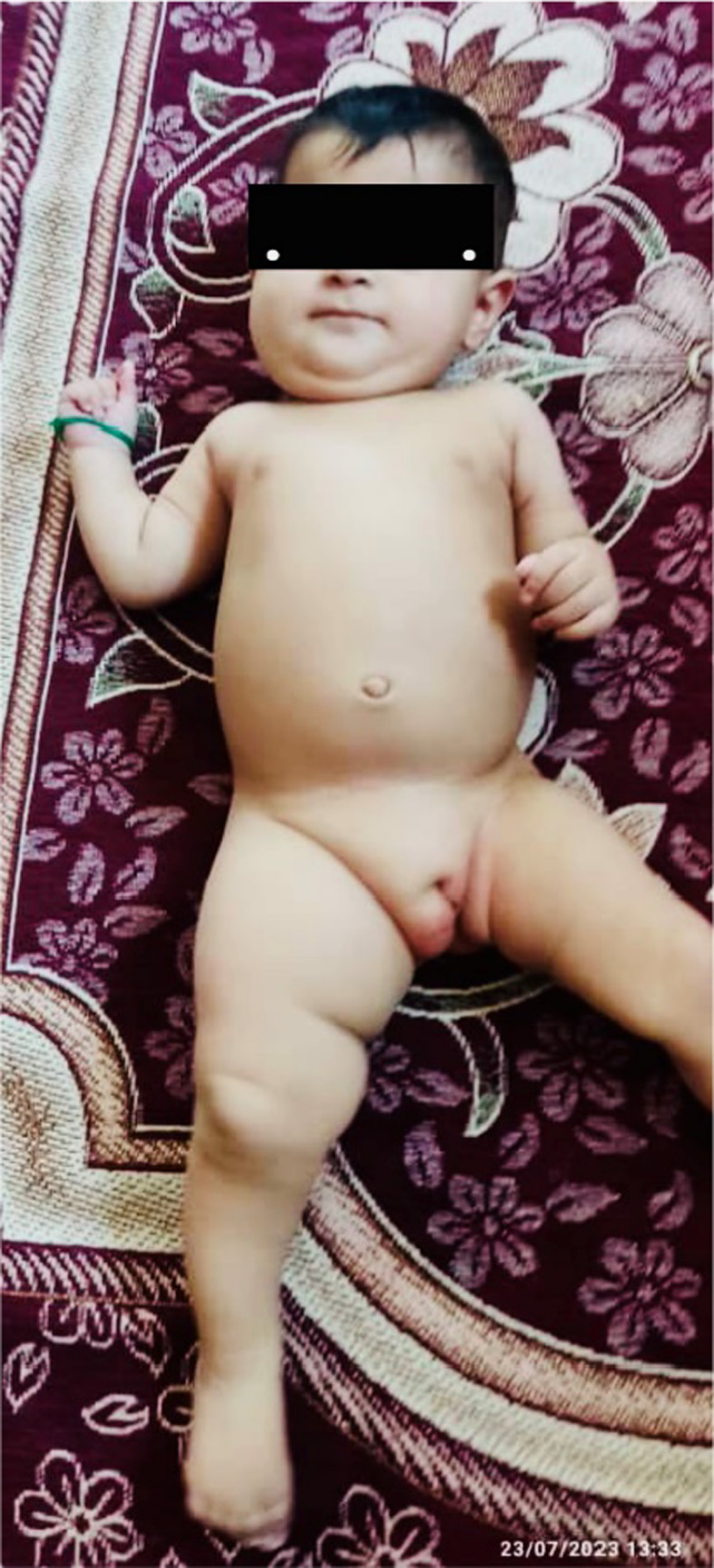
Lipid hemihypertrophy manifested in the right thigh, right labia majora, and below the right mandible.

We monitored the patient until her ninth month of life. No particular treatment was administered (check out Figure 1). Given her age, a surgical operation aimed at extracting fatty tissue was impossible. Instead, we provided her and her mother with nutritional counseling, regularly visited her, and monitored her blood markers, as there was a potential risk of metabolic disorders occurring. Also, we regularly checked the anatomical sites of edema owing to the potential concern of malignancy occurrence.

## 3 DISCUSSION

Hemihypertrophy is characterized by a notable asymmetry between the two sides of the body that exceeds what is to be expected from normal variation. Lipid hemihypertrophy, also known as asymmetric fat distribution, is a rare condition characterized by an abnormal accumulation of fat on one side of the body. This condition can affect various body regions, such as the limbs, trunk, face, or neck.^7^ Instances of other types of hemihypertrophy are as follows: (1) isolated hemihypertrophy: which is a form of hemihypertrophy that is not associated with any genetic disorders, (2) hemihypertrophy associated with overgrowth syndromes: hemihypertrophy is often associated with overgrowth syndromes such as Beckwith‐Wiedemann syndrome, Proteus syndrome, neurofibromatosis Type 1, and mosaic trisomy 8, (3) hemihypertrophy associated with vascular malformations: hemihypertrophy is also associated with vascular malformations such as Klippel–Trenaunay syndrome and Klippel‐Feil syndrome.^8^, ^9^

Understanding the potential problems and disorders that a neonate with lipid hemihypertrophy might encounter until adolescence requires considering the possible complications associated with this condition. One of the primary concerns is the potential for musculoskeletal abnormalities. Asymmetrical growth patterns can lead to limb length discrepancies, scoliosis, or joint problems. These skeletal abnormalities can impact the individual's functional abilities, gait, and overall quality of life. Early assessment by a pediatric orthopedic specialist is crucial for monitoring and managing these potential musculoskeletal issues. It can also cause carpal tunnel syndrome, which is a condition that causes numbness, tingling, and weakness in the hand due to compression of the median nerve. In addition to musculoskeletal concerns, children with lipid hemihypertrophy may also face aesthetic and psychological challenges. The visible asymmetry caused by the condition can lead to body image issues, low self‐esteem, depression, and social isolation. Psychological support from healthcare professionals, including psychologists or counselors, can play a vital role in helping the child cope with these challenges and promoting a positive self‐image.^10^

Another potential problem associated with lipid hemihypertrophy is an increased risk of developing metabolic disorders. Asymmetrical fat distribution can be linked to altered metabolism, leading to insulin resistance, dyslipidemia (abnormal lipid levels), and an increased risk of cardiovascular diseases such as hypertension or dyslipidemia. Regular monitoring of metabolic parameters and early intervention, such as lifestyle modifications and medication if necessary, can help mitigate these risks. Insulin resistance is a condition in which the body's cells become resistant to the effects of insulin, leading to high blood sugar levels. Insulin resistance can lead to type 2 diabetes, which is a chronic condition that affects how the body processes blood sugar. Individuals with hemihypertrophy may be at an increased risk of developing insulin resistance due to the overgrowth of adipose tissue on one side of the body. In addition, lifestyle modifications such as a healthy diet and regular exercise can help manage insulin resistance and prevent the development of type 2 diabetes.^11^, ^12^ Organic acidemias are a group of metabolic disorders that disrupt normal amino acid metabolism, particularly branched‐chain amino acids, causing a buildup of acids that are usually not present. Organic acidemias can cause vomiting, dehydration, lethargy, and seizures. Mitochondrial disorders are a group of disorders in which the ability of the body to generate energy is impaired. Mitochondrial disorders are usually not treatable and lead to a shortened life expectancy. Symptoms of mitochondrial disorders can include muscle weakness, seizures, developmental delays, and vision and hearing problems.^13^, ^14^ Also, it has been associated with an increased risk of developing pheochromocytomas, which are rare tumors that develop in the adrenal glands and can cause high blood pressure, headaches, and sweating.^15^

Furthermore, comprehensive genetic evaluations may be recommended given the association between some genetic syndromes and lipid hemihypertrophy. Genetic testing can help identify any underlying conditions that may coexist with lipid hemihypertrophy. Syndromes such as Beckwith‐Wiedemann syndrome and Proteus syndrome have been associated with asymmetric overgrowth and can present additional health challenges that require specialized management.^16^

Regular monitoring of growth parameters, including height, weight, and body mass index (BMI), is crucial throughout childhood and adolescence. Pediatricians will evaluate the child's growth trajectory, and any deviations or abnormalities in growth patterns can be identified and addressed promptly. Close collaboration between the pediatrician, endocrinologist, and other specialists, when required, can help manage growth‐related concerns effectively. A screening procedure for hemihypertrophy includes two steps: (1) “three measurements – three questions” screening, or assessment of face, palms, and shins, and (2) in‐depth clinical and radiological examination. Surgical management may be necessary in some cases, such as extreme unilateral hemihypertrophy with severe lower extremity manifestations at a suitable age.^17^, ^18^ Continued surveillance for potential neoplasms is additionally a crucial aspect of management. Despite variations in screening recommendations, all emphasize the early detection of abdominal neoplasms, as these are most commonly associated with hemihyperplasia. Screening modalities can encompass physical examinations and assessment for elevation of serum α‐fetoprotein, serum chorionic gonadotropin, and urinary catecholamines (vanillylmandelic acid and homovanillic acid) every 3–4 months until the age of 4. Some scholars propose measuring serum α‐fetoprotein as frequently as every 6 weeks until the age of 4. Since the majority of malignancies originate in the abdomen, it has been posited that abdominal ultrasonography be conducted every 3–6 months until the age of 8 and that clinical surveillance for other neoplasms is contemplated. A complete blood cell count and chest radiography every 12 months until the age of 10 have also been advocated. It is suggested that a complete physical examination be performed at least once every 6 months until growth has been completed.^10^, ^19^

Lastly, it is important to emphasize that each case of lipid hemihypertrophy is unique, and the potential problems and disorders a neonate might encounter until adolescence can vary. Early and ongoing multidisciplinary assessment, intervention, and supportive care are essential for optimizing the child's physical, emotional, and psychological wellbeing.

Reviewing the literature, Leung et al. presented a case of isolated hemihyperplasia, in a 1‐month‐old boy with the asymmetry of the lower extremities (the left lower extremity larger than the right). The size discrepancy was first observed at birth.^10^ In a study by *Stoll* et al., 12 individuals presenting hemihypertrophy were presented. Except for one, all cases were sporadic. The parents were not related. No significant abnormalities were observed in any of the cases' family or pregnancy histories. The diagnosis was consistently made either at birth or in the weeks following birth. The gender distribution comprised five males and seven females, including a mother and her daughter. Hemihypertrophy was localized to the upper limb in one case and to the lower limb in another. One patient exhibited certain characteristics of McCune‐Albright syndrome. Hemihypertrophy was found to be associated with Silver‐Russel syndrome in two patients. In all remaining cases, hemihypertrophy was considered idiopathic. Mental and motor development, as well as puberty, were normal in all cases. As the individuals grew, the bodily asymmetry remained unchanged. Some cases were complicated by orthopedic issues during growth, with scoliosis being the most apparent problem. Limb lengthening was required in two cases. Moreover, one of the patients developed an abdominal tumor. One of the patients had two normal children. Hemihypertrophy is usually not inherited. However, the mother of one of the patients also had hemihypertrophy.^1^ Bou‐Haidar et al. present a clinical case of hemifacial hyperplasia observed in an infant, primarily characterized by lipomatosis and hemihypertrophy of the maxilla. In this case, the manifestation was predominantly confined to lipomatosis, with a notable absence of significant muscular hypertrophy or hyperplasia.^7^ Herein, we presented a novel case of lipid hemihypertrophy which was manifested in the right thigh, right labia majora, and below the right mandible.

## 4 CONCLUSION

In conclusion, hemihypertrophy is a rare congenital disorder that possesses the potential to engender significant asymmetry in the body and lead to functional problems, psychosocial issues, and an increased risk of developing certain tumors. Regular monitoring and screening are essential to detect and manage potential problems and disorders.

## AUTHOR CONTRIBUTIONS

**Kayvan Mirnia:** Supervision; validation. **Maryam saeedi:** Supervision; validation. **razieh sangsari:** Supervision; validation. **Kimia Kazemzadeh:** Investigation; writing – original draft; writing – review and editing.

## FUNDING INFORMATION

None.

## CONFLICT OF INTEREST STATEMENT

The authors declare no conflicts of interest.

## CONSENT

Written informed consent was obtained from the patient to publish this report in accordance with the journal's patient consent policy.

## Data Availability

Data on the case clinical information and images are available from the corresponding author upon reasonable request
The authors declare that they have prepared this article in their “personal capacity” (in other words, “not as an official representative or otherwise on behalf of a sanctioned government”)
